# Automated Impact Damage Detection Technique for Composites Based on Thermographic Image Processing and Machine Learning Classification

**DOI:** 10.3390/s22239031

**Published:** 2022-11-22

**Authors:** Muflih Alhammad, Nicolas P. Avdelidis, Clemente Ibarra-Castanedo, Muhammet E. Torbali, Marc Genest, Hai Zhang, Argyrios Zolotas, Xavier P. V. Maldgue

**Affiliations:** 1 School of Aerospace, Transport and Manufacturing, Cranfield University, Cranfield MK43 0AL, UK; 2 Computer Vision and Systems Laboratory, Department of Electrical and Computer Engineering, University Laval, Quebec City, QC G1V 0A6, Canada; 3 National Research Council Canada, Ottawa, ON K1A 0R6, Canada; 4 Centre for Composite Materials and Structures (CCMS), Harbin Institute of Technology, Harbin 150001, China

**Keywords:** composite materials, impact damage, damage diagnosis, infrared thermography, machine learning, principal component thermography, pulsed phase thermography, thermographic images, support vector machine

## Abstract

Composite materials are one of the primary structural components in most current transportation applications, such as the aerospace industry. Composite material diagnostics is a promising area in the fight against structural damage in aircraft and spaceships. Detection and diagnostic technologies often provide analysts with a valuable and rapid mechanism to monitor the health and safety of composite materials. Although many attempts have been made to develop damage detection techniques and make operations more efficient, there is still a need to develop/improve existing methods. Pulsed thermography (PT) technology was used in this study to obtain healthy and defective data sets from custom-designed composite samples having similar dimensions but different thicknesses (1.6 and 3.8). Ten carbon fibre-reinforced plastic (CFRP) panels were tested. The samples were subjected to impact damage of various energy levels, ranging from 4 to 12 J. Two different methods have been applied to detect and classify the damage to the composite structures. The first applied method is the statistical analysis, where seven different statistical criteria have been calculated. The final results have proved the possibility of detecting the damaged area in most cases. However, for a more accurate detection technique, a machine learning method was applied to thermal images; specifically, the Cube Support Vector Machine (SVM) algorithm was selected. The prediction accuracy of the proposed classification models was calculated within a confusion matrix based on the dataset patterns representing the healthy and defective areas. The classification results ranged from 78.7% to 93.5%, and these promising results are paving the way to develop an automated model to efficiently evaluate the damage to composite materials based on the non-distractive testing (NDT) technique.

## 1. Introduction

In the aircraft sector, polymer matrix composites with reinforcements comprised of carbon fibres, glass fibres, Kevlar, and other materials are commonly employed. For example, carbon fibre-reinforced polymer (CFRP) was originally used in Boeing airplanes in the 1950s [1]. When compared to other materials, the utilization of composite materials in the aircraft industry has increased noticeably over time. The new Boeing 787 Dreamliner is made up of more than half composite materials by weight [2]. Composite materials’ primary advantages are their high specific strengths and stiffness [3]. Composite materials, for example, are stronger and stiffer than aluminium, while being lighter. This weight reduction allows for increased passenger and freight capacity while using less fuel. Corrosion, as well as many other highly reactive chemicals often utilized in aircraft applications, are not a problem for composite materials [4]. Moreover, they are thermally durable and can withstand exposure to extreme weather conditions, such as the vast temperature variations encountered during airplane operations. Another significant benefit of composite materials is their design flexibility. Composite constructions are produced in large single pieces and then cut to their intended final shapes, obviating the need for drilling, bolting, and riveting, which are usually used to assemble metallic structures [5,6].

Infrared thermography, often known as thermal imaging or just thermography, is a non-destructive testing (NDT) technology that has attracted a lot of attention in the last several decades for diagnostics and monitoring [3]. This is mostly due to the fact that commercial infrared or thermal cameras, the primary tool for performing infrared thermography, are constantly increasing in terms of sensitivity and spatial resolution, as well as becoming faster and more affordable. Every year or so, a better camera may be had for roughly the same price as the previous model from the previous year. The same statement may be made about computers, which are needed for control, data processing, and picture display, and which deliver more computational power at a cheaper price year after year. As a result, the range of applications is broadening, from traditional building or electronic component monitoring to more current applications such as artwork inspection or composite materials inspection [7].

Péronnet et al. [8] explored the detection of flaws using an IRT approach. In this study, two infrared thermographic techniques were performed on several types of composites used in the aviation industry: lock-in thermography, and pulse thermography. Nonetheless, testing was conducted on composites with both modest and large thicknesses. When compared to other methods, lock-in thermography was found to have a greater level of accuracy. However, this was ascribed to the wave propagation being optimized, allowing for a better passage through the specimen under test. In addition, Montanini [9] employed lock-in and pulse phase infrared thermography to quantify subsurface flaws in a Plexiglas reference specimen. Thermal pictures captured at various frequencies (frequency domain) were post-processed, and the thermal diffusivity of the material was directly measured. Montanini believed the findings were encouraging and proposed active thermography as a practical, quick, and powerful contactless NDT technology for detecting subsurface faults [10].

A control unit is required to synchronize the energy source with the acquisition system, and a computer system [11] can be used to display and/or process images. In the case of active thermography, signal processing is frequently required to improve contrast and quantification. In general, the reflection configuration is best suited to detect defects close to the heated surface, whereas the transmission configuration allows for the detection of defects close to the rear surface due to the spreading effect of the thermal front. Obviously, the transmission approach is not always simple or feasible. Nonetheless, some specific applications can be found. For example, if the part is hollow, it may be advantageous to use internal stimulation with a liquid (water) or gas flow (air). Because of the delayed arrival of the thermal perturbation, changes in flow temperature (hot to cold or the reverse) allow the detection of abnormal variations in wall thickness or blocking passages in this configuration. However, it should be noted that the defect depth cannot be estimated in transmission mode because the heat front travels the same distance whether a defect is present or not, and regardless of its depth [5].

Recently, thermal deep learning algorithms were used to examine and detect surface and subsurface damage in composite materials [12]. Previous research has shown, however, that traditional machine learning (ML) approaches, such as Support Vector Machine (SVM), still require a pre-processing algorithm to properly handle real-world data in order to overcome the trade-off between overall accuracy and generalization. With this in mind, Erazo-Aux et al. proposed using cubic spine SVM to identify/classify damages in specifically manufactured composites with various health conditions [13]. In high-dimensional characteristic spaces, SVM is a useful tool for ML linear predictors. The high dimensionality of the feature space increases sample complexity and computational complexity. By seeking “substantial margin” separators, the SVM algorithmic paradigm tackles the sample complexity problem. If the entire set of cases is not only on the right side of the separating hyperplane but also far away from it, a half-space separates a training set with a substantial margin. Even though the dimensionality of the characteristic space is vast, limiting the method to produce a significant margin separator could result in a low sample complexity (and even an unlimited one). It should be emphasized that all aspects of the SVM algorithms’ computing complexity, mathematical correlations, etc. are adequately covered in the implementation [14,15]. Different linear and kernel functions (linear or radial), constant widths (σ), and limiting terms (C) are commonly tested when training the SVM model. In contrast to linear SVM models, cubic kernel functions are commonly used in cubic SVM algorithms. This will result in increased classification accuracy in a shorter computational time [16].

## 2. Tested Materials and Samples Features

Two distinct laminates were manufactured with 9 and 18 plies and a ply layup similar to that found in a composite aircraft’s fuselage. The fibre was aligned along the longer dimension of these laminates, which were cut into coupons with diameters of 100 × 150 mm. the coupons were affected using the American Society for Testing and Materials (ASTM) testing process with varying energy levels to produce Barely Visible Impact Damages (BVID).

- Laminate size: 100 × 150 mm
- Theoretical cured ply thickness: 0.18 mm
- Material: UD pre-preg material IMS-977-2.

Low-energy hits cause BVID, which have a dent depth of less than 0.3 mm. A steel hemisphere impactor with a diameter of 13 mm was utilised to create the impact conditions. The conditions listed in Table 1 were used to achieve the necessary impact energies. Two coupons of same thickness were impacted with 8 J impacts to determine whether equivalent damages were induced.

**Figure 1:**
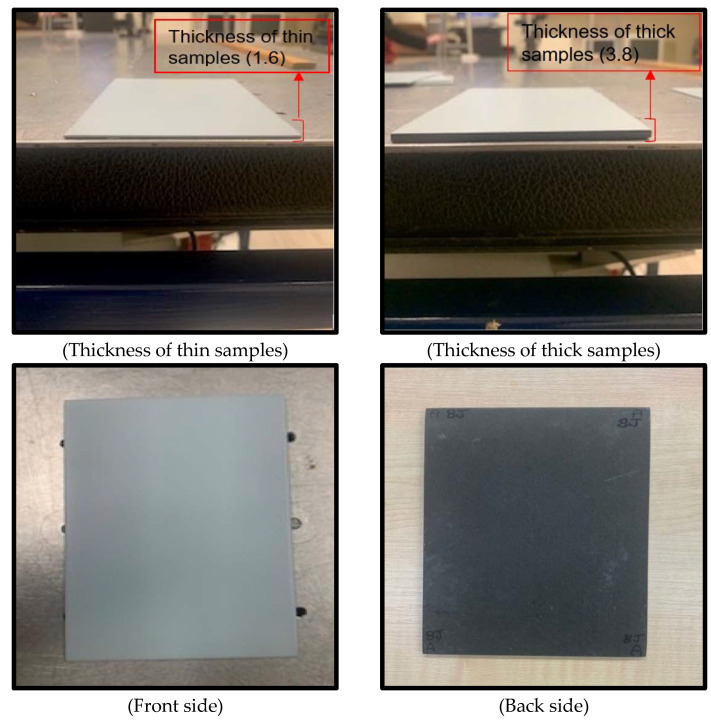
Samples feature.

Table 2 shows the cured thickness of various coupons. The manufacturer’s theoretical per-ply thickness is 0.175 mm. Due to manufacturing variances in coupons and the presence of Foreign Object Defects (FOD) faults in the reference standard specimen, there is a small variation in per-ply thickness. Figure 1 illustrates the thickness of the samples, which are (1.65 mm) for the thin samples and (3.8 mm) for the thick samples. Moreover, Figure 1 also shows the front and back faces as well. However, the proposed approach can be applied on complex (actual) structure geometries. Instead of taking images from one side, images can be taken from multiple sides and applied to the whole procedure (creating machine learning models and getting the results) for each side.

## 3. Lab Components

This section will address the lab components used in the experiments, in addition to the setup and configuration. The lab components are as follows:

- Infrared camera (FLIR X8502sc, Teledyne FLIR LLC, Wilsonville, OR, USA), see Table 3.
- Photographic/Power flashes (BALCAR, Inc. FX60, Teledyne FLIR LLC, Wilsonville, OR, USA).
- Image acquisition software (FLIR ResearchIR Max 4, Teledyne FLIR LLC, Wilsonville, OR, USA).
- Data/pulse generation synchronization (custom-built software/hardware).

## 4. Pulsed Thermography (PT) Method for Image Capturing

During the heat pulse, and during cooling, the infrared camera recorded the thermal evolution on the surface of the inspected CFRP sample for several seconds (about 19 to 20 s) at 50 and 85 frames/s sampling rate. The experiments were carried out in two modes, reflection and transmission, on two faces of the specimen (offering small defect depths from the front face and deeper defect depths from the back face), and each sequence was labelled and stored into its own file.

**Figure 2:**
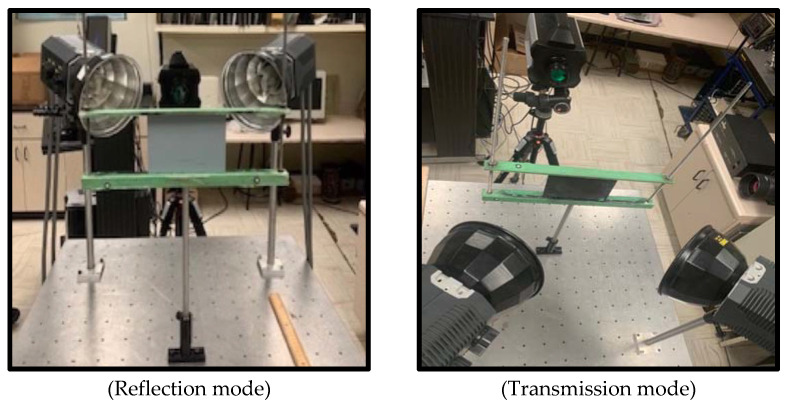
Methods for image capture.

The data source location was as follows: Institution: Computer Vision and Systems Laboratory at Laval University City/Town/Region: Quebec City/Quebec/North America G1V 0A6 Country: Canada. Figure 2 shows the conducted experiments of a pulsed thermographic in different modes.

## 5. Experimental Conduction

The experiments were conducted on 10 different samples, where the first five samples represent the thin samples, while the other samples represent the thick samples. Based on the pulsed thermography method, there are two modes reflection and transmission; based on the reflection mode, 10 sequences were required for each of the thin and thick samples, five images were taken from the front side and five images were taken from the back side. Similarly, based on the transmission mode, five sequences were taken for each of the thick and thin samples. This makes a total of 30 acquired sequences. The following Table 4 summarizes the number of images taken according to the different modes of capture, whether reflection or transmission, for both thin and thick samples.

## 6. Image Processing Methods

Two different methods of image processing have been adopted, and they are as follows:

### 6.1. Principal Component Thermography (PCT) * EOF = Empirical Orthogonal Function

PCT is based on singular value decomposition (SVD), which shows how underlying patterns in a signal can be recovered by projecting the data onto a set of orthogonal basis functions. One of the primary benefits of PCT is that it provides quantitative information about flaw depth. The differences in PPT and PCT results are minor and best discerned through quantitative analysis. However, the PPT and PCT methods outperform the average [17].

### 6.2. Pulsed Phase Thermography (PPT)

Pulse Phase Thermography (PPT) is a technology that combines the benefits of both Modulated Thermography (MT) and Pulsed Thermography (PT). In MT, a single frequency is introduced into the specimen and examined in the stationary regime, whereas in PT, all of the responses are combined into a single transient signal [18]. PPT employs the PT experimental process as well as the MT data analysis technique. It is based on the measured thermal decay T(t) following pulse stimulation using the Discrete Fourier Transform (DFT). The output is represented by a phase function [19,20].

## 7. Image Processing Results of the Pulsed Thermographic Based on Reflection Mode

**Figure 3:**
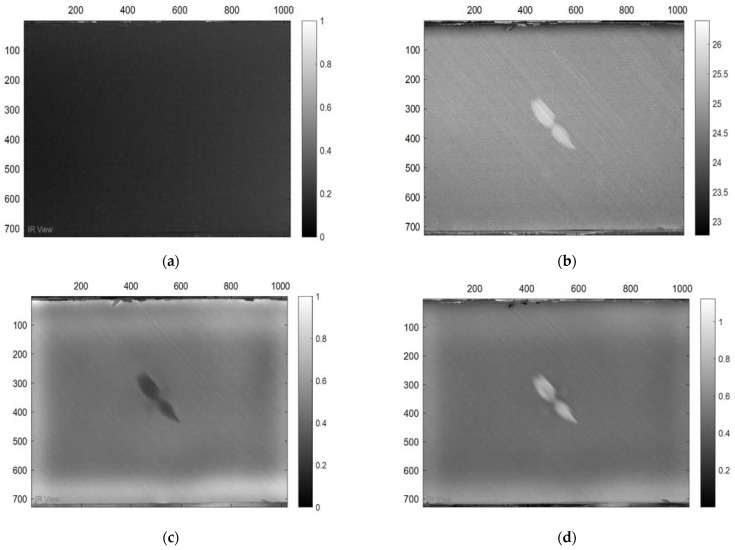
Images Processing Results, (**a**) raw thermogram corresponding to t = 0.8 s. (**a**) Cold image. (**b**) Raw Image. (**c**) BCT Image. (**d**) PPT Image.

**Figure 4:**
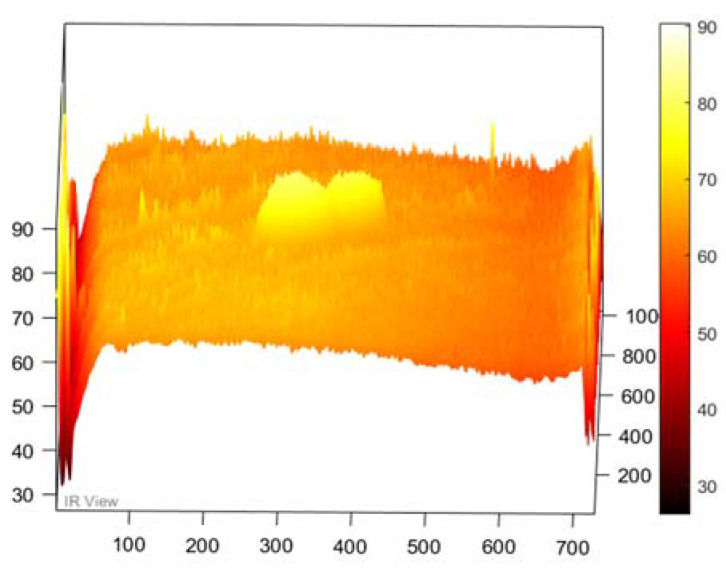
Three-dimensional image, corresponding to PPT.

In this section, the results of the image processing based on the above-mentioned methods (PPT and PCT) are presented, where the final results proved that the tested samples were exposed to damage to varying degrees. Moreover, some damages in the tested samples could not be detected without image processing, which proves the importance of image processing. Figure 3 demonstrates the benefits of image processing, whether PPT or PCT, where the damage can be seen clearly compared to Figure 3a, which reflects the image before exposure to heat. Also, in a three-dimensional image (Figure 4), the damage can be seen clearly where the damage shapes an anomalous pattern on the surface of the 3D image.

**Figure 5:**
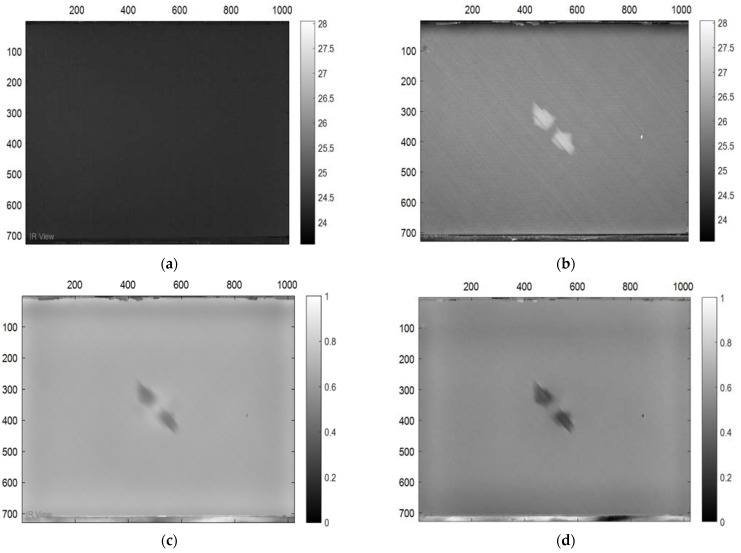
Images Processing Results. (**a**) Cold image. (**b**) Raw Image. (**c**) BCT Image. (**d**) PPT Image.

**Figure 6:**
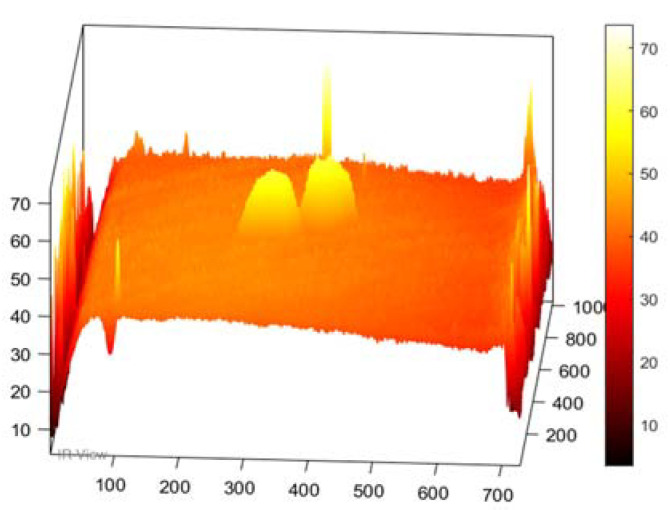
Three-dimensional image, corresponding to PPT.

Also, in the case of the thick samples, the results of the image processing prove the possibility to detect the damage based on both methods (PPT and PCT), see Figure 5. Furthermore, in the three-dimensional image (Figure 6), the damage can be seen clearly on the surface of the samples after the exposure of the sample to heat.

## 8. Image Processing Results of Pulsed Thermographics Based on Transmission Mode

**Figure 7:**
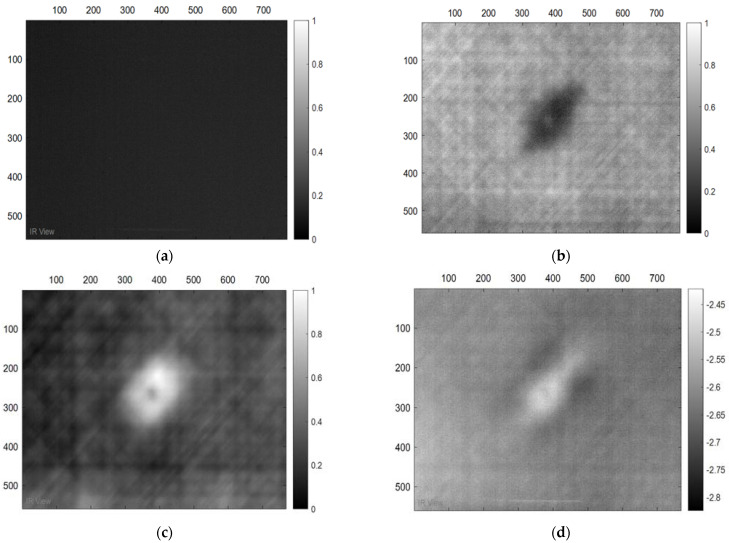
Image Processing Results. (**a**) Cold image. (**b**) Raw Image. (**c**) BCT Image. (**d**) PPT Image.

**Figure 8:**
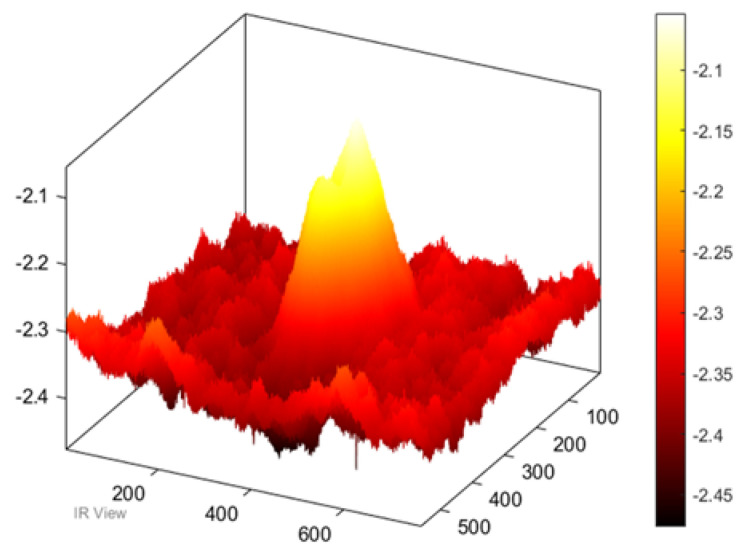
Three-dimensional image of the sample corresponding to PPT.

In this section, the focus will be on the results of the transmission mode instead of the reflection mode (previous section). As shown in Figure 7, the results of the image processing of the thin samples show internal damage on the surface of the tested samples, contrary to the results of the reflection mode, which show details of the surface and shallow damage. Moreover, in the three-dimensional image (Figure 8), the damage can be seen clearly on the surface of the 3D image after processing.

**Figure 9:**
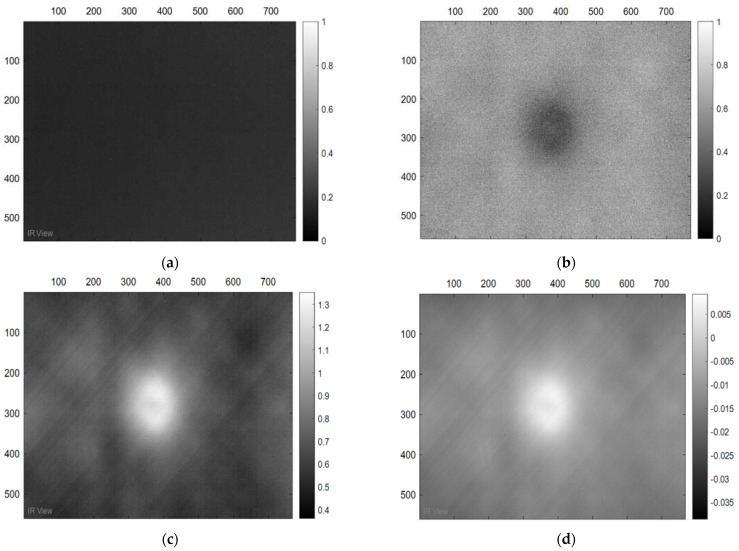
Images Processing Results. (**a**) Cold image. (**b**) Raw Image. (**c**) BCT Image. (**d**) PPT Image.

**Figure 10:**
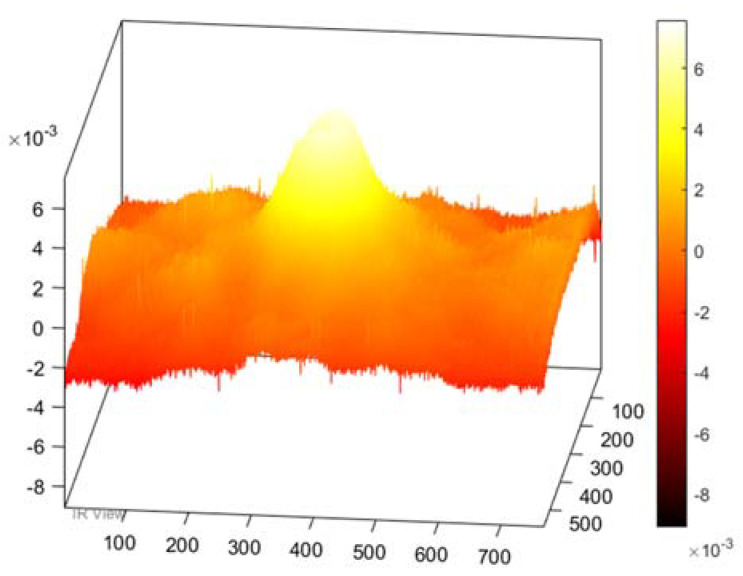
3D of the sample corresponding to PPT.

Another different sample (thick plate) has also been tested based on the transmission mode, and the results show the existence of the damage on the plate surface. Figure 9 demonstrates the final result of the image processing. Also, Figure 10 (3D-image) shows the abnormal pattern of the defective area in comparison to the healthy areas. In this case, damage appears with lesser contrast compared to Figure 7, since the plates are thicker.

## 9. Image Analysis and Damage Detection

**Figure 11:**
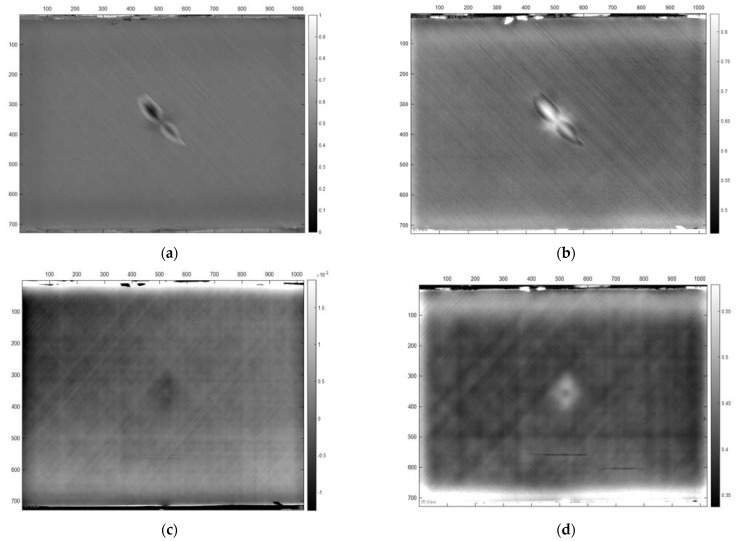
Image Processing Results. (**a**) reflection mood. (**b**) reflection mode. (**c**) transmission mode. (**d**) transmission mode.

After experimenting using the pulsed thermographic method, the outputs of the image processing based on PPT and PCT techniques, the following were observed:

- There is a discrepancy in the raw thermograms because of the different energies to which the sample were exposed.
- Damage contrast varies according to the adopted configuration. i.e., reflection vs. transmission modes, as it is proven that the transmission mode offers more details of internal damage, whilst reflection shows more detail on surface and near to the surface damage.
- The clarity varies in terms of the capturing position in the event of reflection mode or transmission mode. Figure 11 presents comparative results between reflection (a and b) and transmission (c and d) mode.

### 9.1. Statistical Analysis Method Results

One of the methods applied in this study to detect the damaged area in aircraft composite materials was statistical analysis because the thermal imaging technology produces images that include numerical values that can be analyzed. These numerical values, in turn, reflect the shape and structure of the surface that was captured. Therefore, based on the statistical analysis method, it is possible to analyze the numerical values that reflect the shape of the thermal images, and to define a standard values model that reflects the healthy surface and consider any deviation from these values as an indicator of the damaged area.

Based on the statistical analysis method that was followed in this study, seven statistical criteria were applied; three of these criteria have been applied to calculate the range of values using the maximum, minimum, and range of the numerical data, and the other four criteria to calculate the pattern of the numerical values that are reflecting its properties, and these values are: mean, mode, median, and the standard deviation (SD). The following Table 5 summarizes the applied statical criteria.

#### 9.1.1. Statistical Analysis Results of Damage Detection on Thin Reflection Image Sample

**Figure 12:**
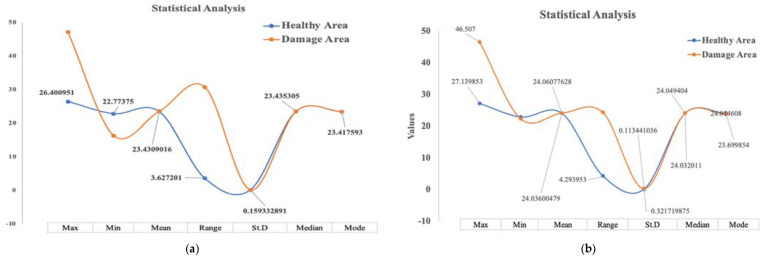

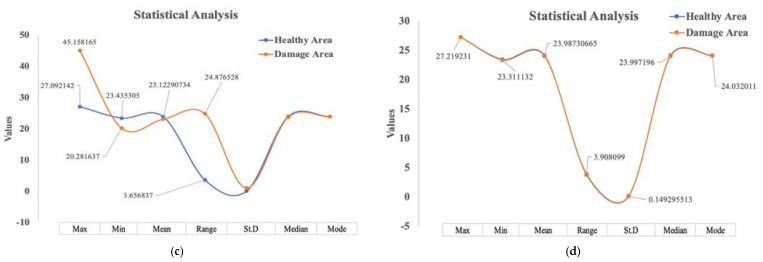
Statistical Analysis results. (**a**) Healthy thin vs. damaged area based on reflection mode. (**b**) Healthy thick area vs. damaged area based on reflection mode. (**c**) Healthy thin area vs. damaged area based on transmission mode. (**d**) Healthy thick area vs. damaged area based on transmission mode.

In this type of thermographic image of thin samples captured based on the reflection mode, the statistical analysis results showed the discrepancy in the final results between the healthy surfaces and the defective surfaces in terms of range rather than in terms of pattern. As shown in Table 6, the maximum value of the healthy surfaces was 26.4, while the value of the damaged surfaces was 47.13. Likewise, the minimum value for the healthy surfaces was 22.8, while the minimum value for the damaged surfaces was 16.4, and accordingly, these values were reflected in the range criterion. On the other hand, concerning the pattern of numerical values representing the image structure, the statistical analysis results showed a great convergence that is difficult to rely on in distinguishing the healthy surfaces from the damaged ones. Therefore, the statistical analysis method for this sort of thermal image captured based on the reflection mode is an effective method. Figure 12a depicts the curve of the statistical values of the healthy and damaged surfaces of the thin sample according to the reflection method.

#### 9.1.2. Statistical Analysis Results of Damage Detection on a Thick Reflection Image Sample

With regard to the statistical analysis results for damage detection on thick samples based on reflection mode, the final results showed the possibility of detecting damage on the surfaces of the samples due to the variation in statistical criteria, whether in terms of range or pattern. As shown in Table 7, the maximum value of the healthy area was 27.14, while the maximum value of the damaged area was 46.16, and the minimum values were 23,43 and 20.28 for the healthy area and the damaged area, respectively. Therefore, this difference between the maximum and minimum values was reflected in the value of the range criterion. In addition, the values of the standard deviation and the mode values were different, as shown in Table 7, and this difference in the values of the statistical results enabled us to distinguish the damaged surfaces compared to the healthy surfaces. Figure 12b visualizes the statistical results.

#### 9.1.3. Statistical Analysis Results of Damage Detection on Thin Samples in Transmission

This section will discuss the statistical analysis results of the numerical values of thermal images of thin samples captured based on the transmission mode. As shown in Table 8, the discrepancy was evident in four statistical criteria, which are the maximum, minimum, and range, as well as the standard deviation, which indicates the possibility of detecting the damaged areas in this sample type based on the statistical analysis method despite the convergence in the other statistical criteria, such as the (mode) value, where the value approximated in the healthy and damaged areas statistically. However, due to the significant difference in the maximum, minimum and range values, we were able to distinguish the damaged areas from the healthy areas. Figure 12c illustrates the statistical results of this case.

#### 9.1.4. Statistical Analysis Results of Damage Detection on a Thick Transmission Image Sample

In the case of thermal images of thick samples that were captured based on the transmission mode, the statistical analysis results were disappointing, as there was no apparent difference in the values of the statistical criteria, neither in terms of range nor in terms of pattern, where the statistical results of the numerical data of the thermal images of the healthy and damaged areas were very close, as shown in Figure 12d. Therefore, it is impossible to rely on this analysis method to detect the damage in the thermal images of thick samples captured in the transmission mode.

In general, the damage detection on aircraft composite materials based on the statistical analysis method for the thermal images, whether for thin or thick surfaces, based on the reflection mode or transmission mode, is an effective method in most cases, except for the last case. Therefore, the statistical analysis method for damage detection in thin or thick samples can be adopted based on reflection and transmission modes, but only for thin samples. Table 9 summarizes the final statistical results.

### 9.2. Machine Learning Detection Method Results

Certain factors can play an essential role in the classification result in terms of probability of prediction accuracy, such as sample thickness and the position in which the data was captured.

In this study, a machine learning method was adopted to detect damage in thermal images of the composite components taken based on the pulsed thermography (PT) method. To apply the machine learning method, three main elements are required: the datasets, the features, and the algorithm required for the purpose of distinguishing between the healthy area and the damaged area. The captured thermogram sequences shown in Table 10 represent the datasets that were trained by machine learning while the properties of the thermal images are represented by the numerical values which reflect the image structure based on the PT method and that will be the features of the datasets.

Finally, the last step is to select the algorithm for predicting the defective area compared to the healthy area. In previous research, several algorithms that separate healthy samples from damaged samples were evaluated, and the Support Vector Machine (SVM) algorithm was identified as the algorithm that provided the highest predictive accuracy 97% [5]. The ideal number of images to have a reliable performance of accuracy is around several thousand images (more or less), according to what was previously evaluated [13], the datasets were normalized for the purpose of applying the machine learning method, as shown in the following table.

As shown in Table 10, 3000 frames have been selected to represent healthy thermal images of thin and thick samples that were captured using two different modes, the first round using the reflection method and the second round using the transmission method. For the healthy thick samples, 2000 frames representing the characteristics of the thermal images using the reflection technique have been captured. Finally, all these datasets were labelled with the number (0), which indicates the characteristics of the healthy samples.

For damaged area, a total of 15,000 frames were generated, where 9000 frames were generated for thin and thick samples using the two different methods (reflection and transmission). Furthermore, only 6000 frames that reflect the characteristics of the thermal images of thick samples collected by the transmission method were generated and uploaded within the training session. Accordingly, the total sum of all healthy and defective samples was 20,000 frames.

Table 11 summarizes the number of observations loaded in MATLAB and their associated features.

As shown in the above table, in the case of healthy samples, the number of observations for the first dataset is 745,472, with 1024 features. Regarding the second dataset, the total number of observations reached 430,080, including 768 features. Regarding the third and fourth datasets, which represent defective samples whether thin or thick, the number of observations reached 2,236,416 and 1,290,240 samples, respectively. The number of features for these observations was 1024 and 768 for the third and fourth datasets, respectively

Based on the training sessions for the datasets, models 1 and 2 were generated based on the results of the Cubic SVM algorithm, and test models 3 and 4 have been exported to test new datasets and ensure the effectiveness of the machine learning method in detecting damage in composite components based on thermographic image processing and the machine learning classification method.

After the data was normalized, it was trained by the classification app in MATLAB, and the Cubic SVM was selected, which was evaluated and proven effective in a previous study with samples that were exposed to artificial damage, while in this study, the samples were exposed to real damage, and the results of the prediction accuracy will be discussed in detail in the following subsections. Table 12 summarizes the characteristics of the machine learning models.

#### 9.2.1. Damage Detection Results of Thin Reflection Image Sample

**Figure 13:**
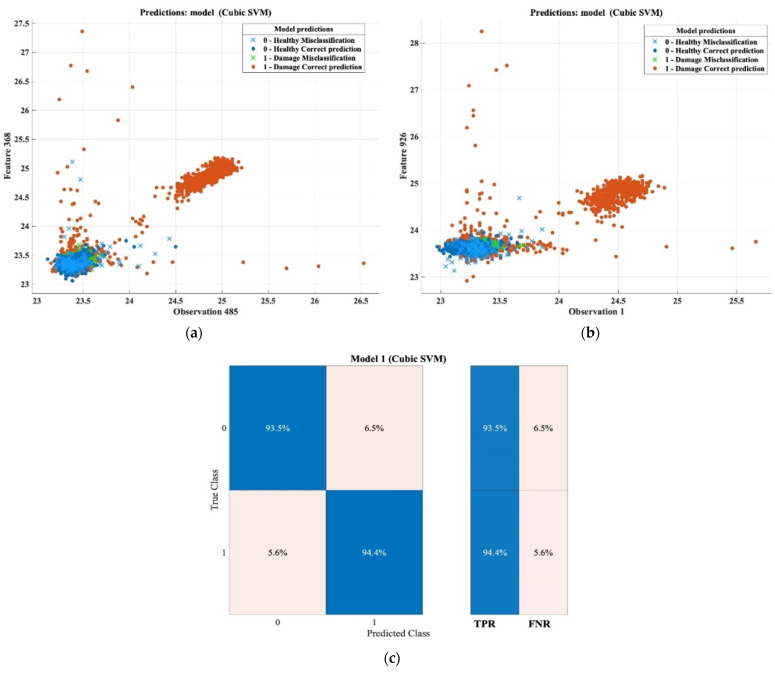
Classification results and confusion matrix for thin samples based on reflection mode. (**a**) Feature patterns of classification results of the cubic SVM model (Observation 485 vs. Feature 368). (**b**) Feature patterns of classification results of the cubic SVM model (Observation 1 vs. Feature 926). (**c**) Confusion matrix.

In reflection mode, the accuracy of prediction in detecting damaged reached 93%, with 189 misclassifications for thin samples. Figure 13 shows the features’ patterns of the thermal images, and the healthy area versus the damaged area. The confusion matrix also shows the final results of prediction accuracy, where the accuracy of predicting a damaged area against a healthy area according to the true positive ratio (TPR) was 93.5%, with a false-negative ratio (FNR) rate of 6.5%, while the accuracy of determining the defective area based on the TPR was 94.4%, and the FNR was 5.6%. Overall, this high rate of detection accuracy proves the effectiveness of this method in detecting damage on the composite components of aircraft based on the pulsed thermography method and the machine learning classification technique, which paves the way toward building an automated approach to damage detection based on non-destructive testing (NDT). Table 13 summarizes the classification results.

#### 9.2.2. Damage Detection Results of Thick Samples in Reflection Mode

**Figure 14:**
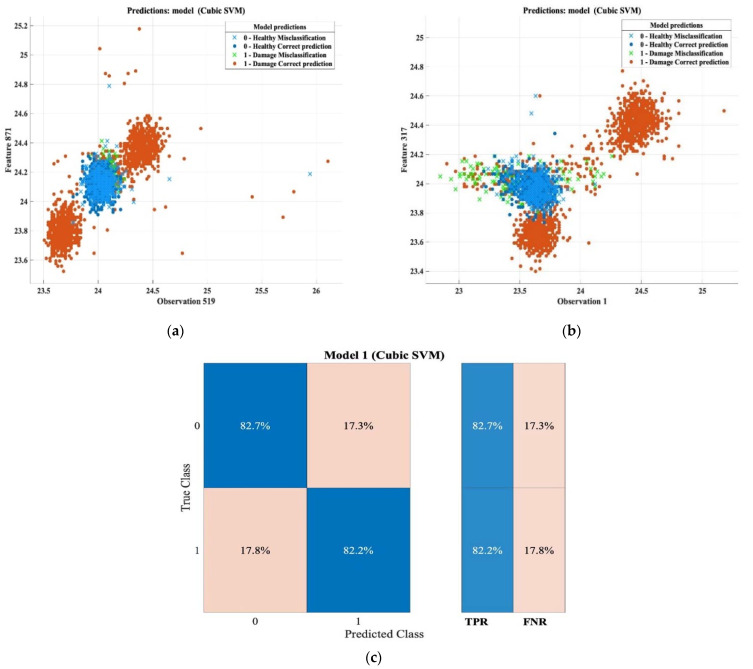
Classification results and confusion matrix for thick samples based on reflection mode. (**a**) Feature patterns of classification results of the cubic SVM model (observation 519 vs. feature 871). (**b**) Features patterns of classification results of the cubic SVM model (observation 1 vs. feature 317). (**c**) Confusion matrix.

Regarding the prediction accuracy for detecting the damaged area in the thick samples according to the reflection method, the accuracy of the detection, in general, was globally 82.1%. Figure 14 depicts the characteristic patterns of the thermal images that were captured. As shown in the confusion matrix, the true positive ratio (TPR) and the false-negative ratio reached 82.2% and 17.8%respectively, and the total misclassification cost was 521. The final results of the training session are presented in Table 14.

#### 9.2.3. Damage Detection Results of Thin Samples in Transmission Mode

**Figure 15:**
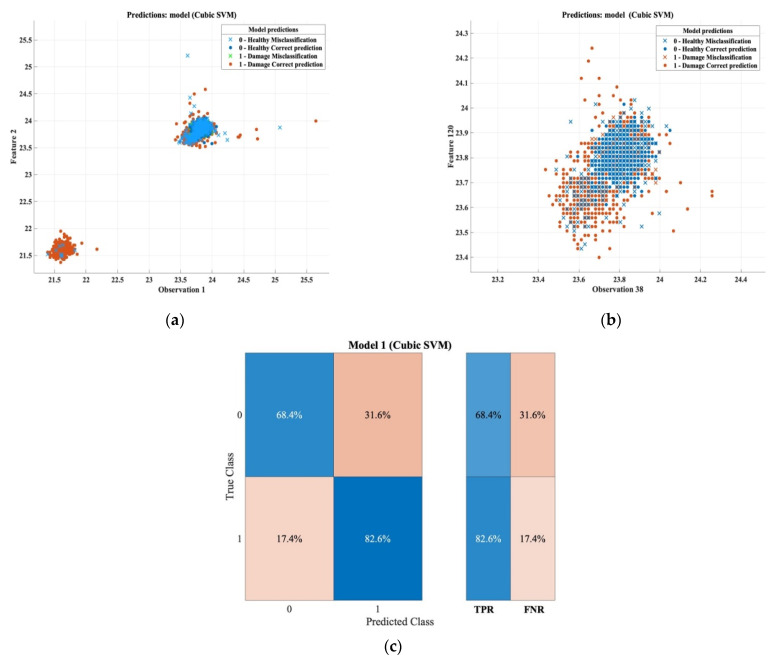
Classification results and confusion matrix for thin samples based on transmission mode. (**a**) Features patterns of classification results of the cubic SVM model (observation 1 vs. feature 2). (**b**) Features patterns of classification results of the cubic SVM model (observation 38 vs. feature 120). (**c**) Confusion matrix.

Moving on to the results that represent the damage detection for the thin samples in transmission, the overall prediction accuracy was 79.4%, the total cost of the misclassification was 462, and, in more detail, based on the confusion matrix, the true positive ratio percentage in determining the defective area was 82.6%, meaning that only 17.4% represents the true negative ratio percentage, which means that 17.4% of the defective surfaces were incorrectly classified as a healthy area. In general, the overall accuracy rate, which is 79.4%, is considered a high percentage that can be relied upon. Table 15 and Figure 15 summarize the final results of the machine learning method in this case.

#### 9.2.4. Damage Detection Results of Thick Samples in Transmission Mode

**Figure 16:**
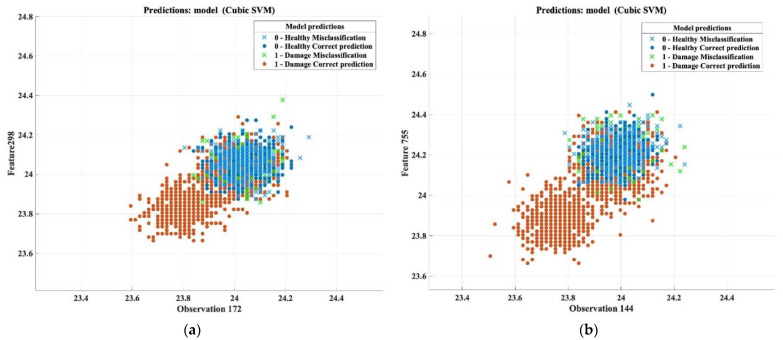

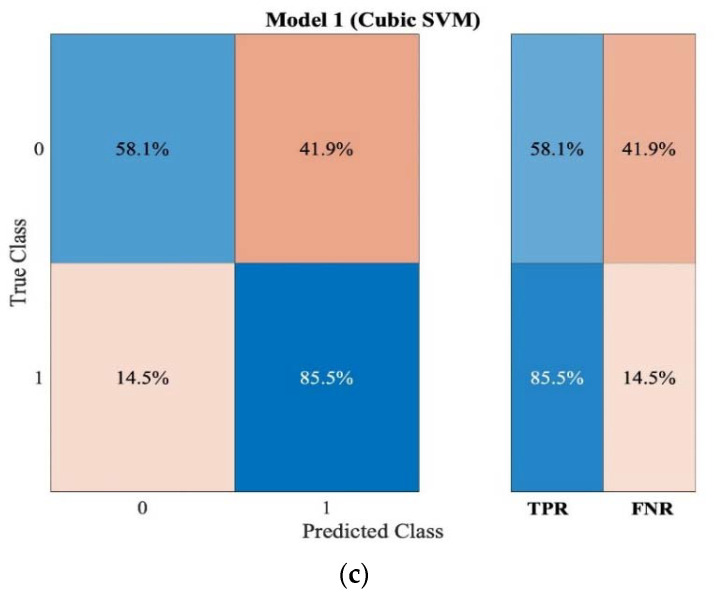
Classification results and confusion matrix for thick samples based on transmission mode. (**a**) Features patterns of classification results of the cubic SVM model (observation 172 vs. feature 298). (**b**) Features patterns of classification results of cubic SVM model (observation 144 vs. feature 755). (**c**) Confusion matrix.

Regarding the results of predicting the damaged area in the thick samples whose thermal images were taken based on the transmission method, the overall prediction accuracy was 78.7%. The following confusion matrix summarizes the ratios of true positive and true negative, where the TPR reached 85.5% in terms of detecting the damaged area, and the FNR percentage was only 14.5%, while the total misclassification cost was 477. The overall accuracy percentage, in this case, was the lowest percentage among other cases, but 78.7% is a good percentage that can be relied upon for detecting the damaged area in the thick composite components based on the transmission method. Table 16 and Figure 16 conclude the final outcomes of the machine learning analysis.

To improve the accuracy classification, especially on the thick samples, the number of the images should be increased by at least by 30%.

In general, and based on the obtained results, implementing the machine learning method to detect damaged areas, whether on the thin or thick samples and whether according to the reflection or transmission methods, is a very effective method. As presented in this section, the result ranged from 78.7% to 93.5%; the variation between these two numbers is due to the capturing mode in which the thermal sequences were taken, in addition to the thickness of the samples used in this study, where the thickness of the samples plays a vital role in the success of the machine learning classification. Therefore, the degrees of classification varied according to the thickness of the samples.

## 10. Conclusions

In conclusion, due to the proliferation usage of composite materials in the field of transportation, especially in aviation, the safety and quality of these materials are essential to preserving human life. Consequently, the need to develop an effective damage detection method is essential. In this study, non-destructive testing technology has been used to detect defects in composite materials, where the pulsed thermography method was used to identify defects in carbon fiber samples used in this experiment. The images were then processed using PPT and PCT techniques. The statistical analysis method utilizing processed data was used first, and the statistical analysis results show that it is an effective method in most cases, except for the last point, which is the thick samples in the transmission mode. Machine learning (Support Vector Machine) was then implemented to detect damaged areas by training significant datasets, obtained from the conducted experiment. It was observed that the presence of defects in the tested samples could be predicted in different proportions according to the thickness of the samples and the approach used (i.e., reflection method or transmission mode). Moreover, the defect detection results in the samples ranged from 78.7 to 93.5%, depending on the classification of the machine learning analysis results. In this study, the reflection mode was more reliable because the crack in the samples was on the surface.

For future work, an automated damage detection model will be developed to increase the effectiveness of this proposed approach. This development will be done by utilizing the data (thermal images) of the actual structure captured by the drone. The data are transmitted and analyzed simultaneously to have a report showing the health of the inspected craft. Furthermore, testing geometry variation of samples to find the classification percentage would be a valuable future work.

## Figures and Tables

**Table 1:** Drop heights and impactor masses for impact testing.

Energy (J)	Drop Height (mm)	Total Impactor Mass (g)
**2.5**	200	1220
**4**	335	
**8**	670	
**12**	1000	
**20**	918	2220

**Table 2:** Layup and thickness of coupons.

Coupons	Layup	Cured Thickness in mm (Averaged)	Per-Ply Thickness
**Impact damage on thin coupons**	[45/−45/90/0/90/0/90/−45/45] (9 layers)	1.65	0.183
**Impact damage on thick coupons**	[45/−45/90/0/90/0/90/−45/45] s (18 layers)	3.8	0.211

**Table 3:** Infrared camera specifications.

Sensor	Image Resolution	Dynamic Range	Acquisition Frequency	Spectral Range
**InSb** **CCD Matrix**	[pixels] 1280 × 1024 used: 512 × 512	[bits] 14	[Hz] 0.0015 to 180 Programmable 50 and 85 during tests	[μm] 3.0 to 5.0

**Table 4:** Experiment conduction.

	Type	Capturing Mode *	Images Frames	Side	No. of Data Sequences	Note
**Samples**	Thin	Reflection	10 × 1000	Front	5	1000 Frames per sequence
				Back	5	
		Transmission	5 × 1000			
	Thick	Reflection	10 × 1000	front	5	
				back	5	
		Transmission	5 × 2000			2000 per sequence

* The capturing method is based on pulsed thermography, which consists of two different modes.

**Table 5:** Statistical Analysis criteria.

Statistical Criteria		Purpose
To find the range of the numerical values of the thermal images	Minimum	To find the lowest value.
	Maximum	To find highest value.
	Range	The find the difference between the lowest and highest.
To find the pattern of the numerical values of the thermal images	Mean	To find the average.
	Mode	The find the most frequent value.
	Median	The find middle value
	Standard deviation (SD)	To measure how spread out the values are.

**Table 6:** Statistical results for the thin samples based on the reflection mode (processed data).

	Max	Min	Mean	Range	SD	Median	Mode
**Healthy Area**	26.400951	22.77375	23.4309016	3.627201	0.159332891	23.435305	23.417593
**Damage Area**	47.127533	16.40193	23.43215487	30.725603	0.164197367	23.435305	23.417593

**Table 7:** Statistical results for thick samples based on reflection mode.

	Max	Min	Mean	Range	St.D	Median	Mode
**Healthy Area**	27.139853	22.8459	24.0360048	4.293953	0.11344104	24.032011	24.014608
**Damage Area**	46.507	22.245995	24.0607763	24.261005	0.32171987	24.049404	23.699854

**Table 8:** Statistical results for thin sample based on transmission mode row thermograms.

	Max	Min	Mean	Range	St.D	Median	Mode
**Healthy Area**	27.092142	23.435305	23.8835772	3.656837	0.08099473	23.892551	23.892551
**Damage Area**	45.158165	20.281637	23.1229073	24.876528	1.05067575	23.752516	23.892551

**Table 9:** Statistical results for thick sample based on transmission mode.

	Max	Min	Mean	Range	St.D	Median	Mode
**Healthy Area**	27.219231	23.417593	24.0912467	3.801638	0.1076612	24.08416	24.08416
**Damage Area**	27.219231	23.311132	23.9873066	3.908099	0.14929551	23.997196	24.032011

**Table 10:** Datasets categories representing thermal image frames.

Cases (Samples × Frames)		Total Frames	Response/Label
**Health area (Dataset 1–2)**	3 × 1000 1 × 2000	5000 Frames	0
**Damage area (Dataset 3–4)**	9 × 1000 3 × 2000	15,000 Frames	1
**Total Trained Frames**		20,000 Frames	

**Table 11:** Data features and number of observations.

Cases	Data Group	Features	Observations
Healthy Area	Datasets 1–2	1024 features 768 features	745,472 430,080
Damage Area	Datasets 3–4	1024 features 768 features	2,236,416 1,290,240

**Table 12:** Machine learning modelling.

	Model	Datasets	Classes	Observations
**Trained Models**	Model 1	Datasets 1 and 3	2	15,120
	Model 2	Datasets 2 and 4	2	18,480
**Test Model**	Model 3	Generated without its trained data as a test model	Cubic SVM Algorithm	New datasets
	Model 4			

**Table 13:** Classification results for thin samples based on reflection mode.

Classification Results	
Accuracy	93.5%
Total misclassification cost	189
Prediction speed	~3100 obs/s
Training time	8.8344 s

**Table 14:** Classification results for thick samples based on reflection mode.

Classification Results	
Accuracy	82.1%
Total misclassification cost	521
Prediction speed	~2800 obs/s
Training time	9.8159 s

**Table 15:** Classification results for thin samples based on transmission mode.

Classification Raults	
Accuracy	79.4%
Total misclassification cost	462
Prediction speed	~3700 obs/s
Training time	5.9488 s

**Table 16:** Classification results for thick samples based on transmission mode.

Classification Results	
Accuracy	78.7%
Total misclassification cost	477
Prediction speed	~3100 obs/s
Training time	7.8478 s

## Data Availability

Not applicable.
